# Multiple Mediating Effects of Conflicts With Parents and Self-Esteem on the Relationship Between Economic Status and Depression Among Middle School Students Since COVID-19

**DOI:** 10.3389/fpsyg.2021.712219

**Published:** 2021-07-20

**Authors:** Jaewon Lee, Hyejung Lim, Jennifer Allen, Gyuhyun Choi

**Affiliations:** ^1^Department of Social Welfare, Inha University, Incheon, South Korea; ^2^School of Education, Korea University, Seoul, South Korea; ^3^School of Social Work, Michigan State University, East Lansing, MI, United States; ^4^Integrative Arts Therapy, Dongduk Women's University, Seoul, South Korea

**Keywords:** depression, multiple mediating effects, conflicts with parents, self-esteem, economic status, middle school students

## Abstract

This study explores associations between perceived economic status and depression among middle school students during COVID-19 in the context of conflict with parents and self-esteem. Data were collected in South Korea in the fall of 2020. A total of 328 middle school students were included, and a multiple mediator model was employed to examine the multiple mediating effects. Middle schoolers’ household economic status was negatively associated with their conflict with parents. Conflict with parents was negatively related to middle school students’ self-esteem. Indirect effects of perceived economic status *via* conflict with parents were significantly associated with depression. The indirect effect of perceived economic status *via* both conflict with parents and self-esteem was related to depression. Government subsidies should temporarily be expanded to improve households’ economic status to potentially improve middle school students’ depression and to enhance relationships between children and their parents during the COVID-19 pandemic. Further, extra financial support from the government should be focused on poor households with children in order to address family conflict, self-esteem, and depression among middle school students.

## Introduction

Since December 2019, people around the world have experienced negative impacts from the COVID-19 pandemic, including loss of life, illness, and financial and mental health impacts. It is not just adults who have been negatively affected; middle school students, usually adolescents between the ages of 11 and 14, have reported worse depression since the COVID-19 pandemic began (e.g., Magson et al., 2021; Ravens-Sieberer et al., 2021). Prior to the COVID-19 pandemic, researchers have found that middle schoolers’ depression is associated with low economic status, high conflict with their parents, and low self-esteem (e.g., Cho et al., 2001; Orth et al., 2008; Yun et al., 2019). Additionally, since the pandemic, households across the world have been negatively affected economically (Falk, 2020; Wilson et al., 2020), so it is of interest to examine the relationship between household economic status and adolescent depression in the period of COVID-19, particularly as both have potentially been worsened by the circumstances of the pandemic. Further, it is of interest to examine the multiple mediators of conflict between adolescents and their parents, which may have increased due to more time spent together at home due to COVID-19-related lockdown measures, as well as adolescents’ self-esteem.

## Literature Review

### Depression Among Adolescents Since COVID-19

A number of studies comparing depression prevalence among adolescents before and during the COVID-19 pandemic showed increased rates of depression among adolescents in many different countries (Hafstad et al., 2021; Jones et al., 2021; Luijten et al., 2021; Magson et al., 2021; Ravens-Sieberer et al., 2021; Thorisdottir et al., 2021). In a sample of Australian adolescents, there was a significant increase in depression symptoms from 12 months before the COVID-19 pandemic to 2 months into the pandemic (Magson et al., 2021). Icelandic adolescents were also administered a survey in 2016, 2018, and during the COVID-19 pandemic in 2020, and there was an increase in depressive symptoms across all age groups, with the increase worse for girls than boys (Thorisdottir et al., 2021). Moreover, Dutch children and adolescents showed worse depression symptoms in April 2020 than in 2018, controlling for factors, such as age and parental education (Luijten et al., 2021). Clinical levels of depression also increased slightly among Norwegian adolescents from February 2019 to June 2020, and German children and adolescents experienced more mental health problems, including depression, after the pandemic began (Hafstad et al., 2021; Ravens-Sieberer et al., 2021). Additionally, in a systematic review of studies conducted from 2019 to 2021, adolescents around the world exhibited higher rates of depression during the pandemic (Jones et al., 2021). Further, in a sample of adolescents and young adults in the United States, adolescents were more likely to report clinically significant depression symptoms during the COVID-19 pandemic than adults (Murata et al., 2020). Therefore, it is of interest to examine the potential impact of such high and increasing rates of depression among adolescents during the COVID-19 pandemic.

### Household Economic Status Since COVID-19 and Adolescent Depression

The COVID-19 pandemic has negatively affected the global economy (Acs and Karpman, 2020; Jeong et al., 2020; Trading Economics, 2021). Although overall less negatively affected than some other countries, South Korea’s economy has been negatively affected by decreased factory output, a reduction in exports, and rising unemployment rates that hit 20-year highs in December 2020 (Jeong et al., 2020; Jackson et al., 2021; Trading Economics, 2021). Further, three studies have examined how adolescents with lower socioeconomic status particularly have been affected by depression since the COVID-19 pandemic (Luijten et al., 2021; McGuine et al., 2021; Szwarcwald et al., 2021). Data collected in May 2020 from adolescent athletes in the United States showed that as their level of household poverty increased, so did their symptoms of depression (McGuine et al., 2021). Further, less affluent Brazilian adolescents were more affected by sadness, irritability, and sleep problems from June to September 2020 than their more affluent peers (Szwarcwald et al., 2021). Moreover, when examining the influence of parental job loss on depressive symptoms, Dutch children and adolescents whose parents had a negative change in work situation by April 2020 were more likely to report depression symptoms than children whose parents’ work situation remained stable (Luijten et al., 2021). Thus, examining adolescents’ household economic status and particularly whether it has changed negatively since COVID-19 are important factors when examining adolescent depression.

### Parent-Adolescent Conflict Since COVID-19 and Adolescent Depression

Since the COVID-19 pandemic, there is some evidence to suggest that conflict between adolescents and their parents has increased (Russell et al., 2020) and that parent-adolescent conflict and support from and satisfaction with family relationships are associated with adolescent depression (Chen et al., 2020; Magson et al., 2021). Compared to a national sample of parents in the United States from 2011, parents reported more conflict between themselves and their children during the pandemic (Russell et al., 2020). Further, in a sample of adolescents living in Wuhan, China, during a COVID-19-related lockdown, higher perceived parental rejection and overprotection, including parent-adolescent conflict, were associated with increased depression (Chen et al., 2020). Additionally, among Australian adolescents, increased conflict with fathers – but not mothers – during the COVID-19 pandemic moderated change in adolescents’ depressive symptoms from before the pandemic to 2 months into the pandemic (Magson et al., 2021).

Moreover, there is some evidence from prior to the COVID-19 pandemic to suggest that parent-adolescent conflict is indirectly associated with adolescent depression through adolescent self-esteem (Portes and Zady, 2002; Barber et al., 2003; Lin et al., 2008; Siyez, 2008; Ozdemir, 2014). In a longitudinal study of Spanish-speaking adolescents in the United States, parent-child conflict and adolescent depression were inversely associated with adolescent self-esteem (Portes and Zady, 2002). In another sample of Taiwanese adolescents, both lower self-esteem and higher family conflict predicted adolescent depression in a logistic regression analysis (Lin et al., 2008). Further, in a sample of Turkish adolescents aged 14–18, conflict with their parents explained 8% of the variance in self-esteem score, while conflict with their parents and self-esteem together explained about 26% of the variance in their depression score (Ozdemir, 2014). Moreover, in a study of Turkish high school students, if adolescents perceived high conflict within their families, they were more likely to report low self-esteem and high depression (Siyez, 2008). Last, in a sample of African-American adolescents, adolescent self-esteem partially mediated the relationship between quality of the parent-adolescent relationship and adolescent psychological functioning (Barber et al., 2003). Thus, more research is needed to examine parent-child conflict since the pandemic and its relationship with adolescent self-esteem and depression.

### Adolescent Self-Esteem and Depression Since COVID-19

One consequence of the COVID-19 pandemic and associated lockdowns and social distancing measures has been a decrease in adolescent self-esteem and an associated increase in depression (Rossi et al., 2020; Pizarro-Ruiz and Ordonez-Camblor, 2021). In Spain, children and adolescents were not allowed to leave their homes except for emergencies from mid-March to late April 2020, and during this time, a survey was conducted to examine the impact of such confinement on young people’s wellbeing (Pizarro-Ruiz and Ordonez-Camblor, 2021). One such outcome was a decrease in self-esteem, with girls showing lower self-esteem than boys (Pizarro-Ruiz and Ordonez-Camblor, 2021). Moreover, although no studies were found examining adolescents specifically since COVID-19, in line with the hypothesis that self-esteem serves as a buffer against negative mental health outcomes, results showed that self-esteem mediated the relationship between fear of COVID-19 and depression (Rossi et al., 2020).

### The Current Study

There have been a large body of research examining the effects of COVID-19 on society (e.g., Falk, 2020; Wilson et al., 2020). However, few studies have addressed pathways related to middle school students’ depression since COVID-19. As middle school students are still maturing and greatly influenced by their family relationships (Greenberger and Chen, 1996; Sheeber et al., 1997; Tucker et al., 2003; Sajjadi et al., 2013; Yun et al., 2019), it is important to account for family factors when considering their depression. In particular, many households have encountered economic challenges due to COVID-19-related economic recession (Acs and Karpman, 2020; Jackson et al., 2021; Trading Economics, 2021). Thus, households’ economic status and family relationships, which are affected by economic status, are important predictors influencing middle school students’ depression since COVID-19. Further, self-esteem is also a critical factor that affects middle school students’ developmental processes and is related to depression (e.g., Rossi et al., 2020). Therefore, it is necessary to investigate how self-esteem influences middle school students’ depression. However, despite the importance of the influence of family factors and self-esteem on middle school students’ depression, little is known about these relationships during COVID-19. During the period of the pandemic crisis, middle school students’ depression might be more influenced by social and psychological mechanisms, including family relationships and self-esteem. Even though those factors are critical to deeply understand depression among middle school students, little is known about these relationships since the COVID-19 pandemic. Thus, this study explores the associations between perceived economic status and depression among middle school students during COVID-19 in the context of a multiple mediation model. We examine the following research questions: (1) Is economic status related to depression among middle school students? (2) Does economic status influence middle schoolers’ conflict with parents and their self-esteem? and (3) Is there a multiple mediating effect of conflict with parents and self-esteem on the association between economic status and depression among middle school students?

## Materials and Methods

### Participants and Study Setting

As the current study addresses depression among middle school students in South Korea, middle school students who were registered in a public school at the time of our study were the target population. Middle school students living in Gyeonggi Province, which is the most populous province in South Korea, were asked to participate in a survey. Data collection took place from September to October of 2020. To avoid face-to-face interviews due to coronavirus, we conducted an online survey by using an official communication tool provided by the middle schools. Parents, students, and teachers can mutually communicate through this tool, which all public middle schools use. The online survey in this study was delivered to middle school students through this tool. We used Google Forms to create the online survey and a link. Before posting a consent form and statement of this research to the communication tool, the online survey questionnaires were refined by experts, including a middle school teacher, in order to protect human rights and reduce misunderstandings of each item. The final questionnaires took about 20 minutes to complete, and respondents received $2 gift card as a reward for their participation. A total of 354 middle school students engaged in the online survey, but 26 students were excluded for the analysis because they declined to participate in the online survey. As we distributed two consent forms to both students and parents, parents were also asked whether they allowed their children to participate in the study. Some of the excluded sample included students whose parents refused their participation. Thus, 328 respondents were included in the final sample. The average age of middle school students in this study was 14.4 years old (average international age = 13.4 years old). Slightly more than half of all participants were girls (55%). As the current study does not include any identifiable information, the Institutional Review Board approved this study (#200810-1A).

### Measures

#### Depression

Depression among middle school students was measured by the Center for Epidemiologic Studies Depression Scale (CES-D; Radloff, 1977). In this study, we used a short-form version of the CES-D with seven items (Santor and Coyne, 1997; Levine, 2013). Each item was rated on a four-point Likert-type scale. Respondents selected one of four response options: 0 = rarely or none of the time, 1 = some or little of the time, 2 = moderately or much of the time, and 3 = most or almost all the time. The specific questions are as follows: “I did not feel like eating; my appetite was poor”; “I had trouble keeping my mind on what I was doing”; “I felt depressed”; “I felt that everything I did was an effort”; “My sleep was restless”; “I felt sad”; and “I could not get going.” For analysis, we used an average score of the seven items and higher scores indicated higher levels of depression. Cronbach’s *α* of the depression variable was 0.86 in this study.

#### Conflict With Parents

Conflict with parents refers to levels of conflict between parents and middle school students during blended learning due to the crisis of COVID-19. This variable consists of four items with a five-point Likert scale. The response options include as follows: 1 = strongly disagree, 2 = disagree, 3 = neutral, 4 = agree, and 5 = strongly agree. The items include the following, all related to blended learning since COVID-19: “I have been scolded more frequently by my parents because they do not like my behaviors”; “I have had increased disputes with my parents because of different viewpoints”; “I feel annoyed about communicating with parents”; and “I do not want to be in the same place as my parent.” The average score of the four items was used for analysis, and a higher score indicates that middle schoolers have experienced more conflict with parents during blended learning since COVID-19. Cronbach’s *α* of this scale was 0.86.

#### Self-Esteem

Middle schoolers’ self-esteem was measured by the Rosenberg Self-Esteem Scale (Rosenberg, 1965). This measure consists of 10 items with a four-point Likert-type scale. Respondents chose one of the following response options: 1 = strongly disagree, 2 = disagree, 3 = agree, and 4 = strongly agree. Self-esteem items included “I feel I am a person with worth, at least on an equal basis with others”; “I feel I have a number of good qualities”; “All in all, I am inclined to feel I am a failure”; “I am able to do things as well as most people”; “I feel I do not have much to be proud of”; “I have a positive attitude toward myself”; “On the whole, I am satisfied with myself”; “I wish I could have more respect for myself”; “I certainly feel useless at times”; and “I sometimes think I am no good at all.” Before analysis, five items were reverse coded. We used an average score of all items, and higher scores indicated higher self-esteem. Cronbach’s *α* of the four-point Likert-type scale was 0.89.

#### Perceived Economic Status

The Leyden Poverty Line suggested by Kapteyn et al. (1988) was used to measure perceived economic status. Using this scale, middle school students’ perception of their household’s economic status was measured. This measure had one question, “In your circumstances, do you consider your household’s economic status to be good or bad?” Respondents were given six response options: 1 = very bad, 2 = bad, 3 = insufficient, 4 = sufficient, 5 = good, and 6 = very good. A higher score on this variable indicated that the middle school student perceived themselves to have a higher economic status.

#### Control Variables

Gender, age, academic performance, and absence of a caregiver after school were included as control variables in this study. Respondents reported their academic outcomes with the following response options: A, B, C, D, and F. Further, middle school students were asked whether they have a caregiver after school, rather than being on their own.

## Analysis Strategy

A multiple mediator model suggested by Preacher and Hayes (2004, 2008) was employed to examine the multiple mediating effects of conflict with parents and self-esteem on the relationship between perceived economic status and depression among middle school students during the COVID-19 crisis. As the multiple mediator model is conducted based upon bootstrapping, it allows researchers to include more than one mediator. Thus, this study accounts for conflict with parents as a first mediator and self-esteem as a sequence mediator. The effects of perceived economic status on depression during COVID-19 were calculated through the multiple mediators. The PROCESS macro 3.4 was used to identify the multiple mediating effects.

## Results

Table 1 shows descriptive statistics of the current study. Average self-reported academic performance among middle school students was a C grade, and 57% of middle school students did not receive care from a parent or other caregiver after school. Middle schoolers’ average depression score was 0.91. On average, they considered their household’s economic status to be sufficient. The average scores of the conflict with parents and self-esteem measures were 2.17 and 1.80, respectively.

**Table 1:** Sociodemographic and psychosocial characteristics of participants.

Variables	% or Mean (*SD*)	Range
Depression	0.91 (0.73)	0–3
Perceived economic status	4.00 (0.90)	1–6
Conflict with parents	2.17 (1.03)	1–5
Self-esteem	1.80 (0.74)	0–3
Age	14.42 (0.71)	12–16
Gender (girl)	55%	–
Academic performance	3.11 (1.03)	1–5
Absence of a caregiver after school	57%	–

Girls (*n* = 182); absence of a caregivers after school (*n* = 176).

Middle schoolers’ household economic status was negatively associated with their conflict with parents (*β* = −0.25, *p* < 0.001; Table 2). That is, middle school students in wealthier households were less likely to report conflict with their parents. As shown in Table 2, conflict with parents was also negatively related to middle school students’ self-esteem (*β* = −0.22, *p* < 0.001). Further, girls tended to have lower conflict with their parents and lower levels of self-esteem as compared to boys (*β* = −0.26, *p* < 0.05; *β* = −0.17, *p* < 0.05). Academic performance was positively related to self-esteem (*β* = 0.15, *p* < 0.001).

**Table 2:** Direct effects of perceived economic status on conflict with parents and self-esteem.

Variables	Conflict with parents	Self-esteem
(Constant)	4.68 (1.17)	2.64 (0.82)
Perceived economic status	−0.25 (0.06)^***^	0.01 (0.04)
Conflict with parents		−0.22 (0.04)^***^
Age	−0.11 (0.08)	−0.05 (0.05)
Gender (girl)	−0.26 (0.11)^*^	−0.17 (0.08)^*^
Academic performance	0.03 (0.06)	0.15 (0.04)^***^
Absence of caregiver after school	0.19 (0.11)	−0.10 (0.08)

* *p* < 0.05;

*** *p* < 0.001.

**Figure 1:**
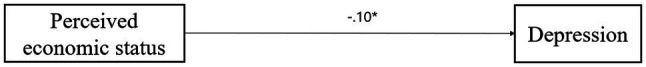
Total effect of perceived economic status on depression.

**Figure 2:**
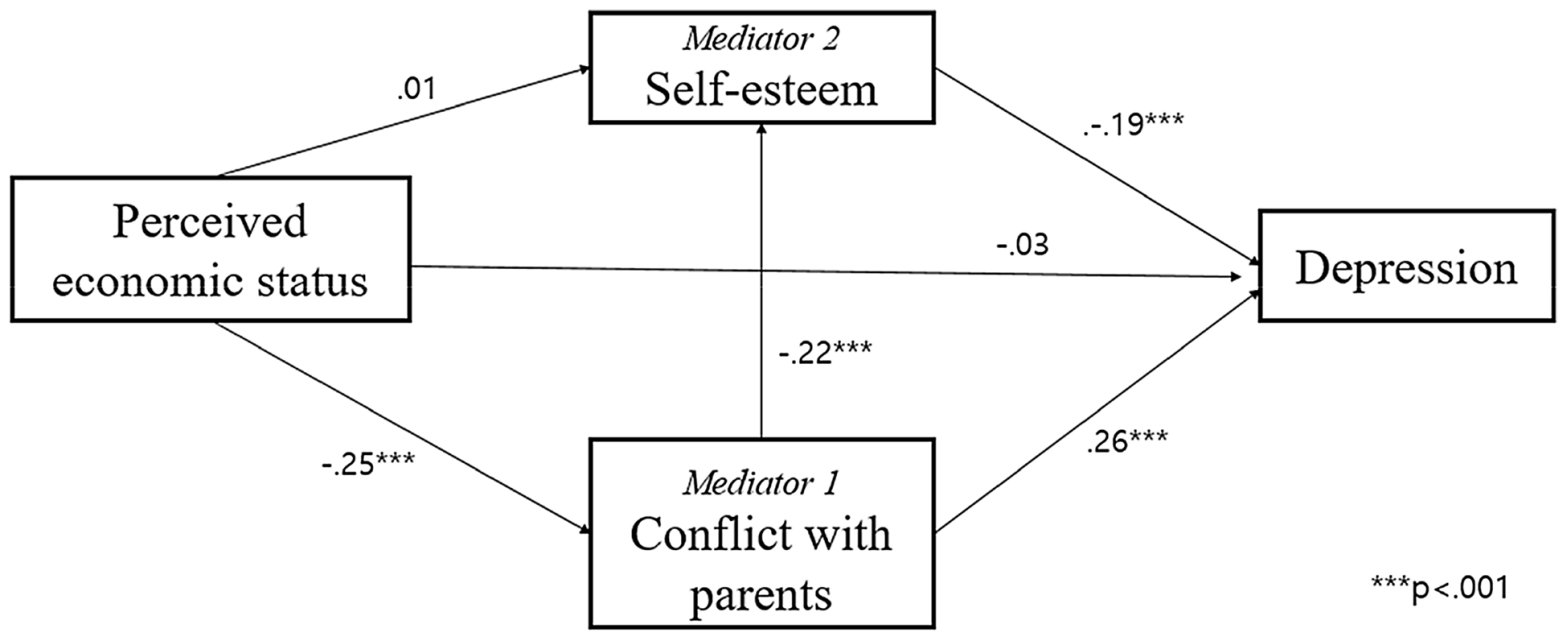
Multiple mediating effect of conflict with parents and self-esteem.

Figures 1, 2 show a multiple mediation model to identify an underlying association between perceived economic status and depression among middle school students since COVID-19 by including two mediators: conflict with parents and self-esteem. Further, model 1 in Table 3 indicates the total effect of economic status on depression, and model 2 shows the direct and indirect effects of economic status on depression after including two mediators – conflict with parents and self-esteem. The total effect of economic status on depression is presented in Figure 1, which was statistically significant in model 1 of Table 3 (*β* = −0.10, *p* < 0.05), and Figure 2 indicates direct and indirect effects after considering the two mediators. The direct effect of perceived economic status on depression was not significant after entering the two mediators (model 2 of Table 3). Conflict with parents and self-esteem were significantly related to depression, respectively (*β* = 0.26, *p* < 0.001; *β* = −0.19, *p* < 0.001). Indirect effects of perceived economic status *via* conflict with parents were significantly associated with depression (*p* < 0.01), while the indirect effect of perceived economic status *via* self-esteem was not significant. Moreover, the indirect effect of economic status *via* both conflict with parents and self-esteem was related to depression (*p* < 0.05). In other words, the multiple mediating effects of conflict with parents and self-esteem on the relationship between economic status and depression among middle school students were significant.

**Table 3:** Direct and indirect effects of perceived economic status on depression using SPSS process.

Variables	Depression	
	Model 1	Model 2
(Constant)	−0.38 (0.82)	−1.29 (0.76)
Perceived economic status	−0.10 (0.05)^*^	−0.03 (0.04)
Age	0.13 (0.06)^*^	0.15 (0.05)^**^
Gender (girl)	0.04 (0.08)	0.09 (0.07)
Absence of a caregiver after school	0.20 (0.08)^*^	0.12 (0.07)
Academic performance	−0.08 (0.04)	−0.06 (0.04)
**Mediators**		
Conflict with parents		0.26 (0.04)^***^
Self-esteem		−0.19 (0.05)^***^
**Indirect effects**		
Perceived economic status -> Conflict with parents		−0.06 (0.02)^**^
Perceived economic status -> Self-esteem		−0.00 (0.01)
Perceived economic status -> Conflict with parents -> Self-esteem		−0.01 (0.01)^*^

* *p* < 0.05;

** *p* < 0.01;

*** *p* < 0.001.

## Discussion

Beyond adults’ depression, depression among middle school students has been given attention in the literature as many have suffered from depression due to school life or relationships with their parents (Greenberger and Chen, 1996; Sheeber et al., 1997; Tucker et al., 2003; Sajjadi et al., 2013; Yun et al., 2019). Since COVID-19, middle schoolers have reported more frequent depression and higher levels of depression (Barendse et al., 2021; Magson et al., 2021; Ravens-Sieberer et al., 2021). As COVID-19 has changed various aspects of our lives, there may be complicated relationships influencing middle school students’ depression. Despite the importance of middle school students’ depression since COVID-19, few studies have addressed their depression in the context of conflict with their parents and their self-esteem. Thus, the current study revealed how perceived household economic status influences depression among middle school students by considering the multiple mediators of conflict with parents and self-esteem. This study found that perceived economic status was related to middle school students’ depression and conflict with their parents. In addition, an indirect effect of conflict with parents was significantly associated with the relationship between perceived economic status and depression. This study also indicated that an indirect effect of perceived economic status *via* multiple mediators, including conflict with parents and self-esteem, was significant. This demonstrated that perceived low economic status influenced more conflicts with parents, which then leads to low self-esteem among middle school students. Those who had low levels of self-esteem were also more likely to be at greater risk of depression during the period of the COVID-19 crisis.

This study revealed that those who perceived themselves to have low economic status were more likely to experience higher levels of depression and conflict with their parents, which is consistent with previous studies (e.g., Smetana and Gaines, 1999; Cho et al., 2001; McLaughlin et al., 2011; Sajjadi et al., 2013). A large body of research has addressed these relationships; however, this study reported that the associations were also significant among middle school students since COVID-19. During the COVID-19 crisis, many households have suffered from economic challenges because of economic recession and job insecurity (Falk, 2020; Wilson et al., 2020). Even if their parents’ income has been negatively affected, middle schoolers have been expected to access additional online educational resources, *via* a laptop, tablet, iPad, or smartphone, which are expensive. Middle school-aged adolescents may be particularly susceptible to peer influence of what electronics to which they should have access, and so middle schoolers who come from families that cannot purchase expensive electronic tools for education and communication in the COVID-19 environment may become depressed because they feel as if they cannot communicate with their friends. Further, parents may have been forced to reduce their spending, including on their children, due to COVID-related economic issues, which may influence their children’s risk of depression.

Likewise, middle school students whose parents have suffered from economic difficulties since the pandemic may be reluctant to communicate with their parents because they feel that their parents’ support is not sufficient, leading to more conflicts with their parents. Further, parents might show more aggressive attitudes toward their children as they encounter more stresses from financial problems since COVID-19. As such, perceived low economic status may critically influence depression among middle school students and conflicts with their parents. Thus, government subsidies should temporarily be expanded to improve households’ economic status to potentially improve middle school students’ depression and to enhance relationships between children and their parents during the COVID-19 pandemic.

Moreover, this study revealed an indirect effect of conflict with parents on the association between perceived economic status and depression. However, the direct effect of perceived economic status on depression was not significant. This implies that it is critical to examine the underlying association between perceived economic status and depression during the COVID-19 pandemic crisis in the context of relationships between children and their parents. Thus, our findings indicated that poor self-perceived household economic status does not directly influence middle school students’ depression, while it does lead to more conflicts with parents, and these greater conflicts result in higher depression among middle school students since COVID-19. This aligns with previous research, which demonstrates the relationship between economic status and conflicts between children and parents (Smetana and Gaines, 1999), as well as the relationship between parent-child conflict and children’s depression in adolescence (Greenberger and Chen, 1996; Sheeber et al., 1997; Tucker et al., 2003; Sajjadi et al., 2013; Yun et al., 2019).

Beyond these associations, this study further contributes to understanding middle school students’ depression since COVID-19. Our findings show that middle school students’ depression was not directly influenced by their perception of their parents’ poor economic status since the coronavirus pandemic. This may be because middle schoolers are young and may not recognize their household’s economic status. Along with this, during the period of coronavirus, middle school students have spent more time in the house due to online education, which requires food for breakfast and lunch that may have previously been provided at school and greater economic resources, such as advanced tools for e-learning. If middle schoolers’ needs are unmet because of limited financial supports from their parents, they may be more likely to fight with their parents, especially when forced to spend longer times in the house. Therefore, extra financial support from the government should be focused on poor households with children in order to provide additional vouchers for food and tools for e-learning.

The current study identified the multiple mediating effects of conflicts with parents and self-esteem on the relationship between perceived economic status and depression among middle school students during the period since COVID-19. Economic challenges since COVID-19 – such as unstable jobs, higher unemployment rates, and reduced income (Falk, 2020; Wilson et al., 2020) – have increased parents’ stressors, while extra expenditures needed for e-learning have had a negative impact on the relationship between children and parents. If parents are less able to financially support their children due to economic hardships and children’s needs are not being met, it may lead to more conflicts between children and parents, in part because middle school students are not as mature as to understand the current difficulties their parents are facing. Given that middle school students are in adolescence, a life stage which may increase troubles with parents, low economic status can further instigate conflicts in an already volatile relationship between middle school students and their parents.

Such conflicts with parents may interrupt communication between parents and their children, which is particularly relevant since COVID-19, because middle school students have been often forced to stay at home rather than going to school and socializing with their friends. In other words, parents are one of the closest persons who middle schoolers can talk to in-person about their daily life since COVID-19. As a good relationship between parents and children is associated with higher self-esteem in adolescence (Bulanda and Majumdar, 2009; Keizer et al., 2019), middle school students who reported more conflicts with their parents are more likely to have low self-esteem than their counterparts who have fewer conflicts with their parents. Thus, frequent conflicts with parents may lead to parents exhibiting aggressive behaviors and scolding their children, and these behaviors may be a buffer against adolescents’ developing positive self-esteem. This is particularly important because low self-esteem is inversely associated with depression (Brage and Meredith, 1994; Marcotte et al., 2002; Orth et al., 2008, 2014; Masselink et al., 2018), and this is also true since COVID-19 as shown in this study. As such, multiple mediating effects of conflicts with parents and self-esteem implied that complicated pathways should be examined to deeply understand depression of middle school students since COVID-19. Over one and half years into the COVID-19 era, our daily lives have been dramatically changed and new life patterns have emerged. Thus, this study considered multiple mediators to explain levels of depression among middle school students since COVID-19. Even though perceived household economic status did not directly influence depression, perceived poor economic status during the pandemic influenced conflicts with parents and self-esteem. Therefore, financial subsidies from the government for households with children as well as financial community supports should be expanded to address mental health problems among middle school students.

Although this study sheds light on the relationship between perceived economic status and depression among middle school students in the context of multiple mediators, especially during the coronavirus pandemic, there are several limitations that impact the interpretation of this study’s findings. First, even if middle school students might have similar characteristics based on their developmental processes, traditional values or cultural differences should be considered, as this study includes a sample from South Korea. Second, findings in the current study are limited to the context of the period during the COVID-19 pandemic. Thus, there might be other factors influencing the relationship between economic status and depression among middle school students before COVID-19 or after COVID-19. Third, other factors which were not included in this study, such as peer influence, might be other important factors influencing depression. Due to the already long length of the survey, we could not include other factors. Along with this issue, some factors, such as cultural factors, sense of control, and resilience, were not included in this study. Thus, interpretation may be limited to demonstrate alternative explanations of the association between socioeconomic status and depression in adolescents. Thus, we recommend that future studies should consider more control variables, which might influence depression among middle school students and support alternative explanations. Fourth, this study employed standardized measurements, except for the variable of conflict with parents. It was difficult to find an appropriate scale that considers cultural differences and fits well with students in South Korea. However, we suggest that other sources of parent-adolescent conflicts could be used in future studies to better measure levels of the conflict. Further, the scale of perceived economic status used in this study is highly subjective. This study could not collect information about real economic status because we did not collect data from parents or guardians. However, we acknowledge that an economic status variable based on income, net worth, and poverty is needed to interpret findings based on objective economic status. Fifth, the sample size was calculated by using a sample size calculator with a 5.24 confidence interval and 95% confidence level. The calculated number was 349 participants; however, we suggest that more participants should be recruited to improve the generalizability of findings by utilizing more advanced power analysis or sample size estimation.

## Implications

Poor perceived economic status and other economic challenges since COVID-19 may lead to numerous deleterious impacts for a family. Direct impacts of such economic challenges include decreased income and job insecurity, which negatively influence quality of life. For a family with children, in particular, middle school students who perceive themselves to have low economic status during the pandemic are more likely to face conflict with their parents, to experience low self-esteem due to less interaction with parents, and to suffer from depression associated with low self-esteem. Although perceived economic status was not directly related to mental health among middle schoolers since COVID-19, this study showed the multiple mediating effects of conflicts with parents and self-esteem on the association between perceived economic status and depression. Therefore, it is important to investigate the underlying pathways in the context of individual and family factors. In particular, middle school students who perceive themselves to have a poor economic status tend to be at greater risk for depression, and they might not have sufficient support for their mental health due to COVID-19-related social distancing measures. Thus, temporarily increasing the number of staffs and professionals who can help with the mental health problems of middle school students, particularly those with lower economic status, might be effective to reduce levels of depression among middle school students. In addition, given that multiple factors influence middle school students’ depression during the pandemic, economic supports from government or community sources should also be focused on programs and interventions to improve family relationships by decreasing conflicts between children and parents and to enhance levels of middle school students’ self-esteem, rather than by only providing financial support to families to improve their economic status.

## Data Availability Statement

The raw data supporting the conclusions of this article will be made available by the authors, without undue reservation.

## Ethics Statement

The studies involving human participants were reviewed and approved by the Institutional Review Board of Inha University approved this study (#200810-1A). Written informed consent to participate in this study was provided by the participants’ legal guardian/next of kin.

## Author Contributions

The authors contributed equally to this study. All authors have read and agreed to the published version of the manuscript.

### Conflict of Interest

The authors declare that the research was conducted in the absence of any commercial or financial relationships that could be construed as a potential conflict of interest.

## References

[ref1] Acs G. Karpman M. (2020). Employment, income, and unemployment insurance during the COVID-19 pandemic. Available at: https://www.urban.org/sites/default/files/publication/102485/employment-income-and-unemployment-insurance-during-the-covid-19-pandemic.pdf (Accessed June 18, 2021).

[ref2] Barber C. N. Ball J. Armistead L. (2003). Parent-adolescent relationship and adolescent psychological functioning among African-American female adolescents: self-esteem as a mediator. J. Child Fam. Stud. 12, 361–374. 10.1023/A:1023948029266

[ref3] Barendse M. E. A. Flannery J. Cavanagh C. Aristizabal M. Becker S. P. Berger E.. (2021). Longitudinal change in adolescent depression and anxiety symptoms from before to during the COVID-19 pandemic: a collaborative of 12 samples from 3 countries. PsycArXiv. 10.31234/osf.io/hn7us, [Preprint]. PMC9349954 35799311

[ref4] Brage D. Meredith W. (1994). A causal model of adolescent depression. J. Psychol. 128, 455–468. 10.1080/00223980.1994.9712752, PMID: 7932297

[ref5] Bulanda R. E. Majumdar D. (2009). Perceived parent-child relations and adolescent self-esteem. J. Child Fam. Stud. 18, 203–212. 10.1007/s10826-008-9220-3

[ref6] Chen S. Cheng Z. Wu J. (2020). Risk factors for adolescents’ mental health during the COVID-19 pandemic: a comparison between Wuhan and other urban areas in China. Glob. Health 16:96. 10.1186/s12992-020-00627-7, PMID: 33036622 PMC7545801

[ref7] Cho S. J. Jeon H. J. Kim M. J. Kim J. K. Kim U. S. Lyoo L. K.. (2001). Prevalence and correlates of depressive symptoms among the adolescents in an urban area of Korea. J. Korean Neuropsychiatr. Assoc. 40, 627–639.

[ref9] Falk G. (2020). COVID-19 pandemic’s impact on household employment and income. Available at: https://crsreports.congress.gov/product/pdf/IN/IN11457 (Accessed May 19, 2021).

[ref10] Greenberger E. Chen C. (1996). Perceived family relationships and depressed mood in early and late adolescence: a comparison of European and Asian Americans. Dev. Psychol. 32, 707–716. 10.1037/0012-1649.32.4.707

[ref11] Hafstad G. S. Saetran S. S. Wentzel-Larsen T. Augusti E.-M. (2021). Adolescents’ symptoms of anxiety and depression before and during the Covid-19 outbreak: a prospective population-based study of teenagers in Norway. Lancet Reg. Health Eur. 5:100093. 10.1016/j.lanepe.2021.100093 PMC8454857 34557820

[ref12] Jackson J. K. Weiss M. A. Schwarzenberg A. B. Nelson R. M. Sutter K. M. Sutherland M. D. (2021). Global economic effects of COVID-19. Available at: https://fas.org/sgp/crs/row/R46270.pdf (Accessed June 18, 2021).

[ref13] Jeong E. Hagose M. Jung H. Ki M. Flahault A. (2020). Understanding South Korea’s response to the COVID-19 outbreak: a real-time analysis. Int. J. Environ. Res. Public Health 17:9571. 10.3390/ijerph17249571, PMID: 33371309 PMC7766828

[ref14] Jones E. A. K. Mitra A. K. Bhuiyan A. R. (2021). Impact of COVID-19 on mental health in adolescents: a systematic review. Int. J. Environ. Res. Public Health 18:2470. 10.3390/ijerph18052470, PMID: 33802278 PMC7967607

[ref15] Kapteyn A. Kooreman P. Willemse R. (1988). Some methodological issues in the implementation of subjective poverty definitions. J. Hum. Resour. 23, 222–242. 10.2307/145777

[ref16] Keizer R. Helmerhorst K. O. W. van Rijn-van Gelderen L. (2019). Perceived quality of the mother-adolescent and father-adolescent attachment relationship and adolescents’ self-esteem. J. Youth Adolesc. 48, 1203–1217. 10.1007/s10964-019-01007-0, PMID: 30887261 PMC6525131

[ref17] Levine S. Z. (2013). Evaluating the seven-item center for epidemiologic studies depression scale short-form: a longitudinal US community study. Soc. Psychiatry Psychiatr. Epidemiol. 48, 1519–1526. 10.1007/s00127-012-0650-2, PMID: 23299927

[ref18] Lin H. C. Tang T. C. Yen J. Y. Ko C. H. Huang C. F. Liu S. C.. (2008). Depression and its association with self-esteem, family, peer and school factors in a population of 9586 adolescents in Southern Taiwan. Psychiatr. Clin. 62, 412–420. 10.1111/j.1440-1819.2008.01820.x, PMID: 18778438

[ref19] Luijten M. A. van Muilekom M. M. Teela L. Polderman T. J. C. Terwee C. B. Zijlmans J.. (2021). The impact of lockdown during COVID-19 pandemic on mental and social health of children and adolescents. Qual. Life Res., 1–10. 10.1007/s11136-021-02861-x [Epub ahead of print], PMID: 33991278 PMC8122188

[ref20] Magson N. R. Freeman J. Y. A. Rapee R. M. Richardson C. E. Oar E. L. Fardouly J. (2021). Risk and protective factors for prospective changes in adolescent mental health during the COVID-19 pandemic. J. Youth Adolesc. 50, 44–57. 10.1007/s10964-020-01332-9, PMID: 33108542 PMC7590912

[ref21] Marcotte D. Fortin L. Potvin P. Papillon M. (2002). Gender differences in depressive symptoms during adolescence: role of gender-typed characteristics, self-esteem, body image, stressful life events, and pubertal status. J. Emot. Behav. Disord. 10, 29–42. 10.1177/106342660201000104

[ref22] Masselink M. Van Roekel E. Oldehinkel A. J. (2018). Self-esteem in early adolescence as predictor of depressive symptoms in late adolescence and early adulthood: the mediating role of motivational and social factors. J. Youth Adolesc. 47, 932–946. 10.1007/s10964-017-0727-z, PMID: 28785953 PMC5878202

[ref23] McGuine T. A. Biese K. M. Petrovska L. Hetzel S. J. Reardon C. Kliethermes S.. (2021). Mental health, physical activity, and quality of life of US adolescent athletes during COVID-19-related school closures and sport cancellations: a study of 13000 athletes. J. Athl. Train. 56, 11–19. 10.4085/1062-6050-0478.20 PMC7863599 33290516

[ref24] McLaughlin K. A. Breslau J. Green J. G. Lakoma M. D. Sampson N. A. Zaslavsky A. M.. (2011). Childhood socio-economic status and the onset, persistence, and severity of DSM-IV mental disorders in a US national sample. Soc. Sci. Med. 73, 1088–1096. 10.1016/j.socscimed.2011.06.011, PMID: 21820781 PMC3191493

[ref25] Murata S. Rezeppa T. Thoma B. Marengo L. Krancevich K. Chiyka E.. (2020). The psychiatric sequelae of the COVID-19 pandemic in adolescents, adults, and health care workers. Depress. Anxiety 38, 233–246. 10.1002/da.23120, PMID: 33368805 PMC7902409

[ref26] Orth U. Robins R. W. Roberts B. W. (2008). Low self-esteem prospectively predicts depression in adolescence and young adulthood. J. Pers. Soc. Psychol. 95, 695–708. 10.1037/0022-3514.95.3.695, PMID: 18729703

[ref27] Orth U. Robins R. W. Widaman K. F. Conger R. D. (2014). Is low self-esteem a risk factor for depression? Findings from a longitudinal study of Mexican-origin youth. Dev. Psychol. 50, 622–633. 10.1037/a0033817, PMID: 23895172 PMC3815504

[ref28] Ozdemir Y. (2014). Parent-adolescent conflict and depression symptoms of adolescents: mediator role of self-esteem. Dusunen Adam 27, 211–220. 10.5350/DAJPN2014270304

[ref29] Pizarro-Ruiz J. P. Ordonez-Camblor N. (2021). Effects of COVID-19 confinement on the mental health of children and adolescents in Spain. Sci. Rep. 11:11713. 10.1038/s41598-021-91299-9, PMID: 34083653 PMC8175710

[ref30] Portes P. R. Zady M. F. (2002). Self-esteem in the adaptation of Spanish-speaking adolescents: the role of immigration, family conflict, and depression. Hisp. J. Behav. Sci. 24, 296–318. 10.1177/0739986302024003003

[ref31] Preacher K. J. Hayes A. F. (2004). SPSS and SAS procedures for estimating indirect effects in simple mediation models. Behav. Res. Methods Instrum. Comput. 36, 717–731. 10.3758/BF03206553, PMID: 15641418

[ref32] Preacher K. J. Hayes A. F. (2008). Asymptotic and resampling strategies for assessing and comparing indirect effects in multiple mediator models. Behav. Res. Methods 40, 879–891. 10.3758/BRM.40.3.879, PMID: 18697684

[ref33] Radloff L. S. (1977). The CES-D scale: a self-report depression scale for research in the general population. Appl. Psychol. Meas. 1, 385–401. 10.1177/014662167700100306

[ref34] Ravens-Sieberer U. Kaman A. Erhart M. Devine J. Schlack R. Otto C. (2021). Impact of the COVID-19 pandemic on quality of life and mental health in children and adolescents in Germany. Eur. Child Adolesc. Psychiatry, 1–11. 10.1007/s00787-021-01726-5 [Epub ahead of print], PMID: 33492480 PMC7829493

[ref35] Rosenberg M. (1965). Society and the Adolescent Self-Image. Princeton, NJ: Princeton University Press.

[ref001] Rossi A. Panzeri A. Pietrabissa G. Manzoni G. M. Castelnuovo G. Mannarini S. (2020). The anxiety-buffer hypothesis in the time of COVID-19: when self-esteem protects from the impact of loneliness and fear on anxiety and depression. Front. Psychol. 10.3389/fpsyg.2020.02177 PMC7683508 33240140

[ref36] Russell B. S. Hutchison M. Tambling R. Tomkunas A. J. Horton A. L. (2020). Initial challenges of caregiving during COVID-19: caregiver burden, mental health, and the parent-child relationship. Child Psychiatry Hum. Dev. 51, 671–682. 10.1007/s10578-020-01037-x, PMID: 32749568 PMC7398861

[ref37] Sajjadi H. Kamal S. H. M. Rafiey H. Vameghi M. Forouzan A. S. Rezaei M. (2013). A systematic review of the prevalence and risk factors for depression among Iranian adolescents. Global J. Health Sci. 5, 16–27. 10.5539/gjhs.v5n3p16, PMID: 23618471 PMC4776790

[ref38] Santor D. Coyne J. (1997). Shortening the CES-D to improve its ability to detect cases of depression. Psychol. Assess. 9, 233–243. 10.1037/1040-3590.9.3.233

[ref39] Sheeber L. Hops H. Alpert A. Davis B. Andrews J. (1997). Family support and conflict: prospective relations to adolescent depression. J. Abnorm. Child Psychol. 25, 333–344. 10.1023/A:1025768504415, PMID: 9304449

[ref40] Siyez D. M. (2008). Adolescent self-esteem, problem behaviors, and perceived social support in Turkey. Soc. Behav. Pers. 36, 973–984. 10.2224/sbp.2008.36.7.973

[ref41] Smetana J. Gaines C. (1999). Adolescent-parent conflict in middle-class African American Families. Child Dev. 70, 1447–1463. 10.1111/1467-8624.00105, PMID: 10621966

[ref42] Szwarcwald C. L. Malta D. C. Barros M. B. D. A. de Souza Junior P. R. B. Romero D. de Almeida W. D. S. (2021). Associations of sociodemographic factors and health behaviors with the emotional well-being of adolescents during the COVID-19 pandemic in Brazil. Int. J. Environ. Res. Public Health 18:6160. 10.3390/ijerph18116160, PMID: 34200307 PMC8201123

[ref43] Thorisdottir I. E. Asgeirsdottir B. B. Kristjiansson A. L. Valdimarsdottir H. B. Jonsdottir Tolgyes E. M. (2021). Depressive symptoms, mental wellbeing, and substance use among adolescents before and during the COVID-19 pandemic in Iceland: a longitudinal population-based study. Lancet Psychiat., S2215-0366(21)00156-5. 10.1016/S2215-0366(21)00156-5, [Epub ahead of print] 34090582

[ref44] Trading Economics (2021). South Korea unemployment rate. Available at: https://tradingeconomics.com/south-korea/unemployment-rate (Accessed June 18, 2021).

[ref45] Tucker C. J. McHale S. M. Crouter A. C. (2003). Conflict resolution: links with adolescents’ family relationships and individual well-being. J. Fam. Issues 24, 715–736. 10.1177/0192513X03251181

[ref46] Wilson J. M. Lee J. Fitzgerald H. N. Oosterhoff B. Sevi B. Shook N. J. (2020). JobInsecurity and financial concern during the COVID-19 pandemic are associated with worse mental health. J. Occup. Environ. Med. 62, 686–691. 10.1097/JOM.0000000000001962, PMID: 32890205

[ref47] Yun J.-Y. Chung H. Sim J.-A. Yun Y. H. (2019). Prevalence and associated factors of depression among Korean adolescents. PLoS One 14:e0223176. 10.1371/journal.pone.0223176, PMID: 31618232 PMC6795486

